# DEFinitely multitasking: Orchestration of petunia floral scent and petal formation

**DOI:** 10.1093/plcell/koaf037

**Published:** 2025-02-14

**Authors:** Linhan Sun

**Affiliations:** Assistant Features Editor, The Plant Cell, American Society of Plant Biologists; Department of Biochemistry and Molecular Biology, The Pennsylvania State University, University Park, PA 16802, USA

The garden petunia (*Petunia hybrida*) is prized for producing large, showy flowers of many different colors. Some cultivars, as well as wild *Petunia* species, also produce an array of volatile organic compounds that make them highly fragrant, a trait that may be underappreciated by humans but is highly favored by hawkmoths and other pollinators. Plant biologists have long been intrigued by the regulation of floral volatile production. Three key transcription factors involved in this process, ODORANT 1 (ODO1), EMISSION OF BENZENOIDS I (EOBI), and EOBII, have been widely studied (Spitzer-Rimon et al. 2012). However, little is known about what initiates the activation of these regulators when the flowers are mature enough for pollination.

In new work, **Dominika Bednarczyk and colleagues (Bednarczyk et al. 2025)** revealed a new regulator of scent production in mature flowers of *Petunia hybrida*, namely DEFICIENS (PhDEF). Surprisingly, *PhDEF* is a MADS-BOX homeotic gene known to determine petal identity in the textbook ABC model of flower development at early stages (Vandenbussche et al. 2004; Rijpkema et al. 2006). However, Bednarczyk et al. found that *PhDEF* expression can also be detected during later stages of flower development, reaching its peak transcript level 1 day post anthesis. More importantly, *PhDEF* exhibited significantly higher transcript levels in the evening compared with the daytime, concurrent with the higher scent emission of petunia flowers in the evening.

The authors used a reverse genetics approach to further examine whether *PhDEF* is involved in volatile production in petunia flowers. As a complete knockout of *PhDEF* resulted in flowers without petals (Rijpkema et al. 2006), the authors chose a virus-induced gene silencing approach to transiently knock down this gene in petunia flowers (Shor et al. 2023). In flowers with suppressed *PhDEF* expression, they observed significantly decreased levels of total volatiles emitted compared with the wild-type controls. Intriguingly, only volatiles derived from the phenylpropanoid pathway were affected by the transient suppression of *PhDEF* expression (Figure). In line with these findings, the transcript levels of major transcription factor genes regulating scent production, including *ODO1*, *EOBI*, and *EOBII*, showed a drastic reduction in *PhDEF*-suppressed petals (Figure). Knockdown of *PhDEF* also resulted in reduced transcript levels of genes encoding biosynthetic enzymes in the phenylpropanoid pathway (Figure) and proteins involved in scent emission. These results pointed to the role of PhDEF in regulating both biosynthesis of phenylpropanoid volatiles and volatile emission at the transcriptional level.

**Figure.**
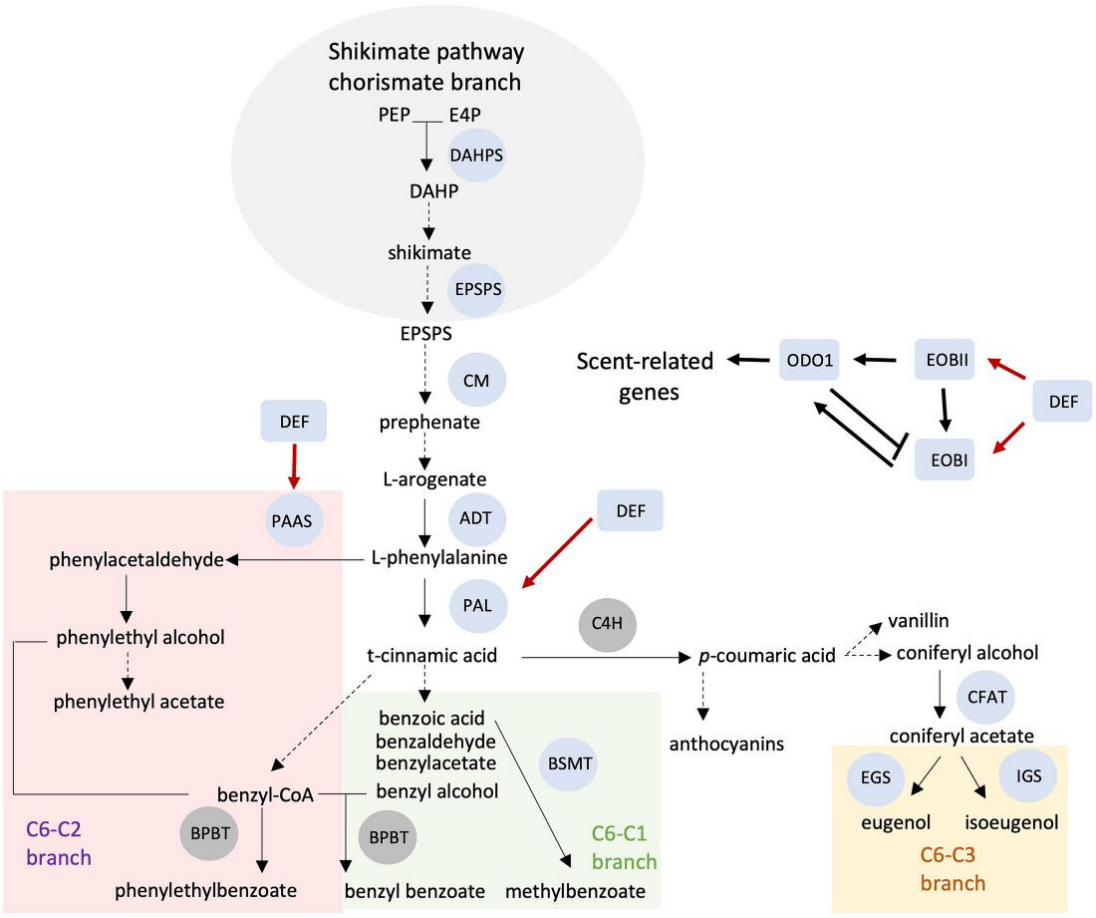
PhDEF regulates scent-related genes. Red arrows indicate genes activated by PhDEF in agroinfiltration experiments. Genes in blue shade exhibited decreased expression in *PhDEF* knockdown petals, and genes in grey shade exhibited no change. Genes encoding transcription factors and biosynthetic enzymes are shown in boxes and circles, respectively. Adapted from Bednarczyk et al. (2025), Figure 5.

Furthermore, the authors used an agroinfiltration system to examine whether PhDEF can activate the promoters of genes encoding scent-regulating transcription factors and enzymes in the phenylpropanoid pathway. Indeed, in both infiltrated petals and leaves, expression of *PhDEF* resulted in upregulation of the transcriptional activity of the promoters examined (Figure). These results further support the role of PhDEF as a transcriptional regulator of volatile production in petunia flowers.

*PhDEF* belongs to the B-class MADS-BOX family, which also includes 2 *GLOBOSA* (*GLO*) genes, *PhGLO1* and *PhGLO2* (Vandenbussche et al. 2004). Expression patterns of *PhGLO1* and *PhGLO2* resembled that of *PhDEF*, but transient knockdown of either gene separately, or both genes together, did not affect volatile production in flowers or the expression levels of scent-regulating genes like *ODO1* and *EOBII*. Therefore, although PhDEF works in concert with PhGLO1/2 in the establishment of flower organ identity at early stages (Vandenbussche et al. 2004; Rijpkema et al. 2006), the role of PhDEF in regulating scent production in mature flowers appears to be independent of PhGLO1/2.

In this elegant work, Bednarczyk et al. (2025) revealed an unexplored link between the classical ABC model of early floral organ identity establishment and later-stage scent production and emission in petunia. As the MADS-BOX homeotic genes are conserved across angiosperms (Rijpkema et al. 2006), it would be interesting to examine whether PhDEF homologs in other plant species are also involved in regulating pollinator-related phenotypes, especially in species with flower-producing volatile profiles distinct from that of petunia for attracting different pollinators.

## Dive Curated Terms

The following phenotypic, genotypic, and functional terms are of significance to the work described in this paper:

EOBII Gramene: EU360893
EOBII Araport: EU360893

ODO1 Gramene: Q50EX6
ODO1 Araport: Q50EX6

EOBI Gramene: AGC00814
EOBI Araport: AGC00814
